# Scrofula; Its Nature, Its Causes, Its Prevalence, and the Principles of Treatment

**Published:** 1847-01

**Authors:** 


### REVIEWS.

---

Art. VII.—*Scrofula; its Nature, its Causes, its Prevalence, and the Principles of Treatment.* By Benj. Phillips, F.R.S. Assistant Surgeon to the Westminster Hospital. Illustrated with an engraved Plate. 8 vo. pp. 355. Philadelphia: Lea & Blanchard. 1846.

That Scrofula is but imperfectly understood, and that the success of the most popular modes of treatment pursued in the disease is far from being satisfactory, is abundantly evident from the eagerness with which the grossest empiricism professing to cure it is embraced, even in the most enlightened communities. The ignorant quack, with his vaunted specific, commands more attention, and often more confidence than the most learned and eminent members of the profession.

Any work, therefore, and especially one based, as that which stands at the head of this article professes to be, upon long and patient observation, comes before the profession with strong claims to its attention and favorable regard. The addition of a single new fact to our limited knowledge of this formidable disease, cannot be deemed a matter of trifling importance. The author of this volume has devoted much time to his subject, and his means of observation, as will be seen from an examination of his work, have indeed been extraordinary. But that the number of facts collected by him has been sufficiently numerous, or that the observations have been made with a care and accuracy, to entitle his labors to such extravagant eulogies as have been passed upon them, we are not prepared to admit. We presume that there is a fashion in literary matters as in all things else under the sun, and that when a book has been cried up by one critic as something extraordinary, the press is too apt to catch the strain and renew the applause, until echo herself grows weary with repeating the sound. Dr. Phillips has produced an excellent work on scrofula; we have read it with great satisfaction, and can cordially recommend it to the profession. At the same time, we are not able to concur in all the conclusions at which the author has arrived, and feel perfectly safe in saying that he has not exhausted the subject. We allow him to speak for himself in the following extract respecting his "own ideas of the nature of Scrofula."

I conceive, then, that Scrofula is a disease of the constitution and that it is most clearly manifested by certain external signs, of which swelling of the subcutaneous lymphatic ganglia is the most conclusive. But *tumid glands*, however they may be situated, are not always a proof that a constitution is scrofulous; they may be the result of local irritation, in an apparently untainted constitution. The glands in the groin may swell, from a sore on the foot; a mesenteric gland may swell under the influence of an ulcer in the intestine; a cervical gland may enlarge under the irritation of teething, or of scalp disease. A *tumid gland*, even *in the neck*, is then no proof that the constitution of the individual in whom it is found is scrofulous. But supposing one, or several cervical glands to become tumid, in the apparent absence of any obvious local irritation, this would constitute a strong ground for suspicion, that the constitution was suffering under the taint of Scrofula. It would not, however, amount to more than suspicion, and the suspicion could scarcely receive absolute confirmation, unless we have the opportunity of observing the contents of the tumor itself. Unless the swelling of the gland be accompanied by the deposit of a product, hereafter to be described, known as *scrofulous matter*, the proof of a scrofulous constitution is, in my judgment, wanting.

In so far then as the local manifestation is concerned, the question, "What is Scrofula?" admits of a tolerably satisfactory solution: but as I regard the local affection, wherever seated, as clear evidence of constitutional disease, it is most important to inquire what that state of the constitution is which causes the local affection; and whether in the absence of the local affection, there be any certain means of determining whether a constitution be scrofulous or not. Because it may be, that the means at our disposal may be powerless to remove the matter when once deposited.

It may be objected to this view of the case, that there are scrofulous diseases in which no scrofulous matter can be de monstrated; "scrofulous ophthalmia," for instance. Supposing such an objection to be made, my answer is this; that the ophthalmia in these cases is simple catarrhal inflammation set up in a scrofulous constitution, and acquiring its particular characters, not in virtue of any thing specific in its nature, but in consequence of the state of the constitution upon which it is grafted. The particular character to which I allude, is a peculiar irritability, which is a prominent feature in inflammation set up in debilitated constitutions, and there is certainly nothing uncommon in such inflammation in persons whose health has been broken by other causes than scrofulous disease. At the time I visited the Whitechapel Workhouse, scrofulous ophthalmia was epidemic among the girls; of eighty-nine present, seventy were suffering from the disease; but of the eighty-nine, only fourteen exhibited any of the ordinary marks of scrofula; and the proportion of girls who presented marks of scrofula, found among those suffering from ophthalmia, was not greater than among those who were free from it.

I believe that diseases regarded as scrofulous, but in which no scrofulous matter is present, are not scrofulous at all, but simply the result of such low inflammatory action as is often set up in a debilitated state of the constitution.

From this it will be seen that Dr. Phillips does not regard "tumid glands, even in the neck," as proof of a scrofulous constitution; but yet in collecting statistics relative to the prevalence of scrofula, he visited parish schools, union houses, prisons, and all other institutions where children were to be found congregated, in order to ascertain how many of the inmates had enlarged glands, cicatrices, or other indications of a scrofulous taint, how many had been sent home for such causes, or how many prevented by them from attending school at all. Now, if tumid glands may exist independently of scrofulous disease, may not these glands inflame and suppurate, leaving cicatrices which could not be told from those of strumous ulcers? What evidence could the author have had that these inmates of public charities, and prisons, were the subjects of scrofula? It is not possible to determine, by mere inspection, whether tumid glands contain scrofulous matter, or pus of another sort; it is not stated that any other means were resorted to in order to determine their nature, and we well know that it is not a very easy matter in every case to make out the diagnosis. Certain mental characteristics, and certain external features have been much depended upon as affording evidence of a scrofulous predisposition. Our author does not attach any importance to these criteria, having found that scrofula invades constitutions marked by every external peculiarity. He says:

The result of my own observation of persons whose constitutions are tainted with scrofula, has satisfied me that there is the utmost possible variety in the external characters of those who present an undoubted scrofulous taint. When the taint is made evident by scrofulous deposits, we find in one case the hair and complexion are dark, in another light; in one the cheeks are rosy, in another pale; in one the alæ nasi are expanded and the upper lip is tumid, in another both of these features present opposite characters. So that it becomes a matter of great difficulty to determine whether the presence or absence of those signs, is most characteristic of a scrofulous constitution.

But it is incumbent on me to endeavor to point out the qualities which are most commonly met with in those who are tainted with scrofula.

In the form of the body there is usually observable a want of muscular development, but even this is often absent. There is often an appearance of plumpness or roundness, which is the result not of muscular development, but simply of an hypertrophied, or infiltrated condition of the cellular tissue, and which rapidly disappears under fatiguing exercise, privation, or disease. Commonly, there is a general paleness and coldness of the surface of the body, which is owing to a feeble circulating apparatus; but in a large number of cases, about one fifth of the whole, that paleness does not extend to the face. The color of the hair is very variable, but for the most part it inclines to a dark tint. Of nearly nine thousand scrofulous children, I have myself examined, a little over 32 per cent. had light hair and eyes. The alæ nasi may be broad, but for the most part they are not so; the upper lip, or even both may be tumid, but in a majority of cases they are not so. There is not, as some persons have supposed, any thing constant in the shape of the lower jaw, or in the appearance of the teeth. The abdomen is commonly tumid. The whole of the mucous surfaces are especially liable to derangement; discharges from the nose, the eye, and the ear, are common. The digestive mucous membrane affords early indications of suffering; the tongue has commonly a dirty whitish coating, the tonsils are usually enlarged, and they are often so tumid as to impress a disagreeable and frequently husky character upon the voice, and to cause snoring when the patient is asleep. A still more deleterious influence is exercised by these tumid bodies; they lessen so much the channel for the passage of the air in respiration that the sufficient development of the chest may be interfered with. The stomach and bowels are frequently disordered, and digestion is ill performed; acrid eructations are common, flatulence is often very troublesome, and the action of the bowels is very irregular, sometimes relaxed, at others constipated; sometimes the evacuations are clay colored, very offensive, and of varying consistency; at others, having a redundancy of bile. Similar evidences of derangement are observed in the air passages, commencing at the nose, (which exhibits increased secretions upon the occurrence of very slight variations in temperature,) and passing through their whole length. Similar phenomena are observed in the mucous tissue of the genito-urinary system; the bladder often shows an impatience of the presence of the urine, and the desire to avoid it is often frequent. The skin, though often dry and hard, is sometimes the seat of a considerable greasy exhalation; sometimes it is found to be fetid and sour. The acidity of the exhalation may be so decided as to determine a reaction upon litmus paper; in many of the cases observed by Mr. Kaye on the Mediterranean coast it was so. The scalp and other parts of the cutaneous integuments are often the seat of eruptive affections. The absence of vascular and muscular energy often causes the child to lie and sit about much, and indisposes him to enter into the energetic games of his playfellows. As to the intellectual development claimed for scrofulous persons, I am bound to say that it is usually wanting. That many scrofulous children present that character is quite true; but the result of very careful observation has convinced me, that the overwhelming majority are without those superior intellectual qualities which have been pointed out as their ordinary character. Among the better classes, the feebleness of a scrofulous child attaches to him an interest which, without it, he might not have enjoyed. To compensate for his physical inferiority, the anxious parent seeks to make him mentally superior to his bodily stronger fellows, and frequently succeeds; but often the limit of healthy action is passed, the nervous and intellectual systems have the vital action concentrated on them too intensely; the sufferer loses flesh, the general health languishes, and the intellectual faculties may give way, destroyed by an opposite but not less sure method than that which breaks down the poor man's child.

If, as our author adds, scrofula be a disease of the constitution, and may exist without being manifested by the ordinary local signs; or if these local signs do not indicate necessarily the existence of a scrofulous constitution, of what value are these observations? They were intended to determine under what circumstances, and in what localities the disease was most frequent; and yet according to the author's own statement it is impossible, in a large majority of cases, to ascertain whether a patient be scrofulous or not. A knowledge of the frequency of any disease presupposes a knowledge of the means of recognising it. Statistics concerning the prevalence of any disease are exceedingly valuable when the disease can be readily recognised. By such data we are often able to arrive at a knowledge of the causes of disease. Thus, we know that intermittent fever originates only at certain seasons, and in particular places, and, therefore, that it is caused by some influence which is only active at such periods and in such localities. If Dr. Phillips had been in possession of means by which scrofula, when existing, could always be detected, and had found it much more frequent among children who were crowded together in large alms-houses, union houses, &c., it would have assisted us very materially in deciding upon the causes of the disease. Since that has not been made manifest we confess that we are not able to perceive how any great amount of good can result from such observations.

We pass over the third chapter, on the scrofulous deposit, without any remark, and reach the next, which is an interesting and valuable one. In this the author treats of the state of the organ in which the morbid product is about to be deposited. That enlarged glands, even in scrofulous subjects, often exist without being the seat of scrofulous matter, and simply in a state of congestion or inflammation, there can be no doubt.

Commonly, if not always, glandular structures do undergo considerable change before scrofulous matter is deposited in them. They acquire a considerable increase in volume, in density, and in vascularity. The bulk may be ten or twenty times what is natural to them, the density may equal or exceed that of veal, and the change of color from increased vascularity is very remarkable.

A point of considerable interest is here presented. Is this state of the gland determined by the circulation within it of blood which has undergone change, or is it independent of the blood? Does the blood fit the organ to receive the deposit, or does the organ fit itself? This is an important question, but of very difficult solution. Important too, with reference to treatment; because if the action set up were purely local, means might be taken to change it, and render it unfit for the deposit. If the action depend only upon a general contamination of the blood, how comes it that all the lymphatic glands are not equally affected? It is notorious that they are not. The bronchial glands are affected more than twice as often as those of the mesentery, the latter four times as often as those of the neck, though in many respects least exposed, and the last named glands four times as often as those of the axilla and the groin. There must be a reason for this. It may be that the cause of the greater frequency in the glands of the mesentery, is the irritation set up in them by depraved chyle, the result of bad food or improper feeding; which state may be kept up until the blood is sufficiently charged with improper materials to occasion a deposit to be made; and I apprehend the cause of the greater frequency of the deposit in the cervical than in the inguinal or axillary glands, is that they are more exposed to alternations of temperature and to irritation; that, therefore, cæteris paribus, under the same amount of constitutional irritation, congestion is more rapidly developed in them, and therefore the deposit is more frequently found in their substance.

The author regards scrofulous matter as a deposit from the blood, without attempting to determine whether it is the consequence of previous alteration in the composition of the blood itself, or of disease in some organ which forms and causes the deposit from healthy blood. He remarks:

As a general law, it is now admitted that morbid products are derived directly from the blood; and the questions then to be resolved are these:—Has the blood previously undergone a change in its elements, the part in which the deposit is made remaining unchanged? Or, does the blood remain in a healthy condition, the part in which the deposit is made exercising a specific influence upon the blood, and determining the character of that deposit? Or, are both removed from a healthy condition before the product is deposited?

In the case of most morbid products, the result of diseased action is to determine the deposit of matter, the previous existence of which in the blood cannot be demonstrated; yet it has been said that tubercular matter has been observed in the circulating blood. In the case of scrofulous matter, no one but Lugol has, so far as I know, asserted the observation of its presence in the circulating mass. His statement is, that he found nodules of such matter, already formed, carried along with the blood. Supposing the observation to be correct, there is no proof that such nodules were formed in the circulating fluid. Bearing in mind that this statement is unsupported by any similar observation made by others, of even the occasional existence of scrofulous matter in the blood, the evidence that I may offer in support of the opinion, that such matter comes from the blood, may be regarded as insufficient. I certainly cannot give a demonstration of the soundness of that opinion. Still, if I show that in the state of health, the blood contains materials such as are found in scrofulous deposits, and that in particular states of the economy, those materials are increased or diminished; and if I show that when particular materials are increased, scrofulous deposits are more frequent, and that the deposits are made in the cellular, or other structure of the organ, and not in the lymphatic channels, I submit that we shall be fully justified in assuming that scrofulous matter is a deposit from the blood.

The analyses of healthy blood, made by Marcet, Berzelius, and Lecanu, correspond so nearly, that we may assume that the elements are correctly determined; and we find them to consist of potash, soda, carbonate and phosphate of lime, albumen, fibrin, and probably gluten. These elements may be found in scrofulous matter, and therefore the notion of its being separated even from healthy blood, would not be a startling proposition to lay down. All that would be required to constitute the scrofulous matter, would be that the elements should be differently combined. But that which makes it improbable that the matter can be formed from healthy blood is, that in the state of health those deposits do not occur; it is therefore fair to infer that there must be a previous change, either in the blood, or in the organs through which the blood circulates, and in which the deposit takes place, or in both, before it can occur.

In the analyses of scrofulous blood by various chemists, our author has not found sufficient uniformity to enable him to determine what changes this fluid undergoes, although in every instance there was found a "considerable deviation from the condition of the healthy blood." He quotes numerous authors, and then gives his own experience, in the following quotation:

The observations of Andral, Gavarret, and others, show that the solid, as well as the fluid constituents of the blood, undergo considerable changes from the influence of food, as well as from that of disease; but it is not shown that the increase or diminution affects all the constituents equally. There may be, at the same time, an increase of one and a diminution of another; those constituents appear to have no mutual dependence on each other.

Enfeebling agents seem to affect, in the first instance, the proportion of red globules; it is constantly diminished, while the proportion of fibrin may be increased. And supposing food to be the agent by which the change is accomplished, the process seems simple enough. Bad food produces bad chyle, bad chyle imperfectly renews the blood, perverts its elements, diminishes, destroys, or augments its plasticity, causes the predominance of certain principles over others, and possibly so changes the composition of the blood, as to determine in it non-analogous productions.

As a general rule under prolonged abstinence, the quantity of blood diminishes, successively, during the whole time of its duration, and at last the quantity becomes so small that one only wonders how it circulates; and in these cases it is commonly found that the proportion of albumen increases, while that of fibrin diminishes.

The imperfect accomplishment of the functions of depuration, as is observed in the Berlin Report, is also a principal cause of morbid states of the blood; any change in the exhalation of carbonic acid gas, or aqueous vapor, in the quantity of sensible and insensible perspiration in the urinary and other secretions, produces alteration in the characters of the blood; and if the materials for those several products be left to accumulate in the blood, they act as poisons; it must be evident, therefore, that any material interruption of those functions, if not compensated for, or supplied by some other, must necessarily induce a change, not only in the quantity or quality of the materials of the blood, but also in its vitality.

To show how far surrounding circumstances, by inducing Anæmia, may alter the natural condition of the blood, I may state, after Andral and Gavarret, that in 1,000 parts of healthy venous blood, we may find:—3.0 fibrin, 127.0 globules, 80. solid matter of serum, 790. water; while in Anæmia its state has been found so changed as to yield: 1.2 fibrin, 36.0 globules, 80 solid matter of serum, 881.8 water. Here the diminution of globules and the increase of water is very striking, so also is the decrease of fibrin.

Baumes stated that in scrofulous patients the different constituents of the blood are less intimately mixed than in healthy persons, the union of the molecules being feebler than in the state of health. Thouvenel observed the same state, and conceived that it was owing to an excess of acrimony. Denis also alludes to it, but he refers to an excess of alkalies. Supposing acrimony to mean acidity, it is certain that both alkaline and acid principles are capable of producing important changes in the constitution of the blood.

Dubois (d'Amiens) also observed the diffluence and imperfect coagulability of the blood in scrofulous persons; he also pointed out a change produced in the form of the globules; he found them "lenticular, the central depression, or spot, being extended beyond its natural limits, and so defined that they looked like wheels," "many of them being also deformed, notched, or otherwise irregular."

I have examined the blood in sixty-seven instances of scrofula, and although I have almost always observed a considerable deviation from the condition of healthy blood, the changes have not presented sufficient uniformity to induce me to regard any particular condition as specially characteristic of scrofula; the changes are such as seem to belong to a tolerably extensive group of affections, all, it is true, being connected with disordered nutrition and debility.

In almost every case, the coagulum was relatively small, the serous menstruum large, the clot was usually very soft, almost diffluent; in a few instances only it was tolerably firm. In almost all cases, the proportion of globules was considerably under the healthy standard. The fibrin had not usually undergone much change; in a few cases it exceeded, in many more it was below the healthy standard. In most instances there was a considerable increase in the proportion of albumen; in almost every instance the proportion of salts was found to exceed the healthy standard; in some instances it was nearly doubled. At one time I thought I had clearly made out, that the proportion of chloride of sodium was deficient, and certainly in many cases it was so; but in many other cases that deficiency was not evident. If succeeding observations should establish a frequent co-existence, it might be well to carry out Russell's salt water plan in the treatment of the disease; he conceived it to be efficacious. It may be, that salt water so used acts as a stimulant of the whole economy, increasing plastic energy, and thus improving the vigor of the system.

In some cases I have observed, under the microscope, as did F. Dubois, that the coloring matter adhered closely to the globule, and that a certain portion of it seemed to be dissolved in the serum, but I do not, with him, regard this as an ordinary character of the blood in such cases. I have usually met with this peculiarity when the blood has been drawn many hours, and I have been more disposed to regard it as a consequence of a disposition to early decomposition than as a proof of any vital change. M. Dubois has always, in such cases, observed the spheroidal and the lenticular globules. He conceived that the lenticular globules were changed in form, some appearing as if perforated like wheels, others irregular or notched. Although I have observed similar appearances in the blood of scrofulous persons, I have also observed such appearances in the blood of persons who did not suffer from scrofula; in fact, in many of those diseases in which anæmia is a distinguishing feature; and I am not, therefore, in a condition to point out any particular state of the blood which is certainly characteristic of scrofula; but it is certain that if we examine the blood when the marks of scrofula are evident, its altered condition is also evident; but I know no particular condition of the blood which clearly marks the existence of scrofula. However the occurrence of similar changes, at the same time, in different regions of the body; seems to point to some other than a local agent, as producing the change in the organ in which the deposit is made—and that agent the blood.

This discrepancy in the analyses of the blood in scrofula may have been owing to the absence of inflammation, or to the different degrees of inflammatory action existing at the time the blood was abstracted. The following are the results obtained by ourselves in the analysis of the blood of a strongly marked scrofulous subject, with considerable enlargement of the cervical and mesenteric glands, the latter of which being also much inflamed:

Temperature of the blood, 98½°

Specific gravity, 1028

| | |
|---|---|
| Water, | 807. |
| Solid residue, | 193. |
| Fibrin, | 3.836 |
| Albumen, | 83.333 |
| Salts and extractive matter, | 20. |
| Blood corpuscles, | 87. |

The fibrin and water are slightly increased in this specimen; the salts are very much augmented, being nearly double the normal quantity, while the corpuscles are somewhat diminished. The few analyses of scrofulous blood which we have made, like those of our author, have not been so uniform in their results, as they have been in fever and some other diseases, and yet, in the different cases, there was sufficient diversity of symptoms to warrant the belief, beforehand, that the results would be various. But unsatisfactory as these observations have been, if we are to make any farther progress in the investigation of scrofula, this must be the starting point. That the blood is changed in this affection is shown by all the observations that have been made concerning it; and although it may be very long before the precise alteration in this fluid which constitutes scrofula is ascertained, yet it may be done by making a sufficient number of analytical observations to enable us to determine the true relation between the pathological conditions of the blood and certain local signs or symptoms. We must ascertain in this way the anatomical characters of the disease in its various stages, and we must learn by well marked signs to recognise the disease in these different stages; we may then hope, by collecting statistics as to the circumstances under which it most frequently occurs, to learn something of its causes.

The author's opinion in regard to the causes of scrofula may be gathered from the following extract:

The alleged causes of scrofula are so many, and their action is said to be so constant and so efficient to produce the disease, and so few human beings can be wholly protected from their influence, that it is wonderful so many persons should appear to be exempt from the affection. It is equally surprising how slender usually is the proof offered by the advocates of a particular cause, in support of its complete efficiency, to induce the development of the disease. The consequence of such vague assumption is, that those who are not satisfied with the sufficiency of one alleged cause, are prepared to advocate as conclusive the influence of another, and it may be a very opposite one, with no stronger evidence in support of the latter theory than was furnished in favor of its predecessor.

One person advocates the opinion that the *hereditary* is the only cause; another contends that the disease is *always acquired*, and never inherited; one regards *contagion* as an efficient cause; another maintains that the disease is never thus communicated. One points to the *bad air of towns* as the cause; another finds the disease more prevalent in the country; one refers the evil to *farinaceous;* another, to *animal food.* It would be easy to enlarge this catalogue, but it will be sufficient to mention, that hereditary influence, syphilis, bad air, bad food, and a cold and damp atmosphere are the causes to which have been most frequently assigned the production of scrofula. The error of each theory is its exclusiveness; and when we reflect upon the difficulty of estimating the unmixed influence of any single cause, and when it is made probable that many causes are in action, we can scarcely comprehend how it happens that able inquirers should maintain, with so much pertinacity, not alone the efficiency, but also the universality of one.

The difficulty of estimating the force of any of the so called causes of scrofula, is owing to the fact that the opportunity of observing a single agent in action alone is very rarely afforded; where one cause exists, another is, almost certainly, intimately associated with it; and to assign to each its proper influence is rarely possible. This is particularly the case with bad food, bad air, and bad clothing; the existence of one almost implies the presence of another. He who is too poor to buy good food, is also too poor to procure good lodging; and although there may be instances where we find much privation associated with the casual occupation of good lodgings, usually it is not so, and we have not had opportunities of observing such cases with sufficient frequency to make any useful deduction from them. As far as it can be accomplished, however, we must attempt to estimate the power of the many influences which are said to be efficient to produce scrofula, because it may be easier to avoid the causes of the disease than to effect its cure when once developed; and therefore if we can indicate the sources of evil with sufficient clearness, much may be done to withdraw the people from their influence.

The patient labor of our author has not resulted in the addition of anything new to our curative resources in this most formidable disease. He seems, indeed, to have attached far more importance to statistics relative to its prevalence, to the refutation of popular theories, and to determining the occupations or modes of living which favor or prevent scrofula, than to the remedial measures demanded when the disease is once fully developed. He divides the treatment into preventive and curative. Under the first head will be found many valuable hints as to the proper method of raising children, so as to guard against the disease. Indeed, all that is said upon this subject is eminently practical and important. An ounce of prevention is better than a pound of cure, is a maxim in which he seems fully to believe, and in few diseases, we are persuaded, can more be done in the way of prophylaxis, and less in the way of cure than the one under consideration. We find, therefore, no cause of quarrel with Dr. Phillips, because he lays as much stress upon the hygiene as upon therapeutics of scrofula. One of the preventive measures suggested, and by far the most efficient, he is not able to sanction. Lugol, in the true utilitarian spirit, has proposed to put restrictions upon marriage, thus limiting the propagation of the species to those who enjoy sound and vigorous health. He would not pitilessly sacrifice children, as the Spartans did, because they were too feeble to become useful to the State, but he would "arrest the evil at its source, by interdicting marriage to sickly and infirm persons." Doubtless this would be drying up one most prolific source of human suffering, but would it not be at a great sacrifice of social and domestic happiness? Such is the conviction of society, as well expressed by our author in the following paragraph:

Lugol's proposal to establish legislative restraints upon marriage, and thus render health and vigor necessary qualifications for wedded life, may be less repulsive to the humane tendencies of modern civilization, than were the stoical principles of Spartan government; but his suggestions would be as little tolerated in the social condition of our times, as would the code of Spartan laws, and we must look to other means than these, for preventing or arresting the development of scrofula.

What are some of these? They are given in this volume in detail and with great judgment. Diet is one of the most influential.

Every child should derive its first food from the breast of a healthy woman; but many children cannot obtain this supply. The mother may be weakly, but if she has milk, she often chooses to nurse her child; and if she flags, from the drain upon her, she fortifies herself to meet it, by the use of as large a quantity of stimulants as may be necessary to support her failing strength; but such feeding often deteriorates her milk and lessens its nutritive power, so that the child's nutrition suffers. Or, the mother's supply may be cut off, then, if the circumstances of the parent admit of it, a foster-mother may be obtained, and the evil be thus partly remedied. But a foster-mother may not be, indeed, by the many, is usually not, attainable, and artificial feeding must be substituted. We have already shown, that under even favorable circumstances, that is objectionable. But when driven to it, the object we must seek to obtain is, to place the child, as nearly as may be, in the same condition as it would be if suckled at the mother's breast.

The fluid which mostly resembles woman's milk, is that of the ass; but the supply is so small, that, practically speaking, it cannot enter into our consideration as a substitute for the milk of the mother. The ordinary substitute for the mother is the cow; and we have already seen how greatly her milk differs from that of woman. It may be diluted, but though richer than woman's milk, in certain materials, it is poorer in others; and if dilution reduces one element to its proper standard, it leaves another below it; and the milk of the cow, even then, continues to be very unlike the child's natural food. Further, it usually happens that many cows concur to furnish food for a single child; the milk of twenty, or even fifty cows, may be mixed together, and although the milk of one cow may not disagree with the child, the mixture of two or more may. Besides, before the child gets the milk, it has usually been drawn many hours, often long enough to undergo a complete separation into parts, and it is no longer a homogeneous fluid, like that which the child draws from the mother's breast. Before it is used, it is often subjected to a heat sufficient to coagulate the albumen, and is thus rendered more difficult of assimilation. Those evils might, to some extent, be remedied, though in practice they will usually continue. The milk may be obtained from a single cow, may be properly diluted, and may be given before any separation has taken place.

Exercise ranks next after food, and is indispensable to the development of the youthful system, and to the maintenance of health. Good air and nutritious food are necessary, but they will not avail without the free, full exercise of the body, which causes digestion and an active circulation. Dr. Phillips conveys the idea in forcible language in the subjoined extract:

It is not, however, enough to provide the means for making the blood pure by the influence of good food and good air; but means must be taken to make it circulate through the body with the necessary vigor. This it is which gives the value to the games of children, whereby active exercise is provided. Three things are therefore necessary to produce perfect health in a child, even when born of the ordinary vigor: good food, good air, good exercise. Give the child these, and no matter what may be the ailments of its parents, or the climate in which he lives, you will do much to build up a vigorous and healthy man. In England it is among the rural population that the conditions to which I have alluded are most fully realized, in as far as concerns air and exercise; but after the child has been deprived of the mother's breast, its food is almost exclusively vegetable. Taken as a class, it is amongst this portion of our population that life has the highest numerical value; but it is among them that scrofulous swellings are most commonly found.

If we had room we should like to extend our extracts on this head, but for other topics connected with the prevention of scrofula, we must refer to the work itself.

Of the curative treatment laid down in this volume, we are not able to speak so favorably. It strikes us as, in some points, essentially defective. It indicates, indeed, all the popular remedies in use in the treatment of scrofula, and gives an estimate of their relative value; but yet there is a deficiency of detail which will be sensibly felt by the young practitioner. Moreover, the preparatory treatment so important to success in the management of this disease is not as fully detailed as we should have liked to have seen it. In a word, the author does not appear to have dwelt with as much satisfaction upon this branch of his subject as upon some others, perhaps because his faith in the remedial treatment is not great. We close our notice of his work by quoting his remarks relative to iodine as a remedy in scrofula:

In my own practice, I have exhibited every form of iodine extensively in cases of scrofula, and supposing the patient to remain exposed to the influence of the same conditions in which the disease was at first manifested, and the period of the year to be that which has not been found favorable for the cure of the disease under other modes of treatment, I cannot say that I have had reason to estimate the curative powers of iodine so highly as many others have done. I know that among the out-patients of hospitals, whose circumstances remain unchanged, and who apply at the latter end of autumn, or the beginning of winter, we may often exhibit iodine in every form for weeks or months, without producing any sensible amelioration in the patient's condition. I know also that at the beginning of summer, a patient similarly affected and similarly treated, will, often in a few weeks, exhibit a marked improvememt—but how much of this should be referred to iodine? How much to season?

I by no means wish to express the opinion that iodine has no curative influence in scrofula; and although I believe that it is not, ordinarily, strong enough to make head against the disease in an unfavorable season of the year, yet I think I have known some cases in which decided benefit has seemed to result from its use, even when the season and other circumstances have not been favorable, and when no change in those circumstances has occurred, beyond the exhibition of iodine; and yet, even then, I refer the good to a general alterative action upon the economy, and not to any specific action; the general health has improved under the employment of the medicine, and the local disease has abated. Such cases have, however, formed a minority of those in which iodine has been administered by me, and I have endeavored, though not satisfactorily, to account for these exceptional cases, by some change, some effort made by the system itself.

What the exact influence of iodine is in scrofula, it is difficult to determine—I mean when not administered in combination with other substances than potass. I am satisfied, however, that in many cases under the influence of iodine, the tongue will become much cleaner, the appetite will improve, and the secretions will acquire a healthier character. And the impression left on my mind is, that the good which may be experienced from the use of this medicine, is not owing to any specific influence which it exerts over scrofula, but to its occasional power of modifying the mucous surfaces, so as to enable them to assist in producing healthy nutrition.

Whatever good may be derived from iodine when uncombined, I think that when associated with particular substances, with iron, for instance, its power over the disease may be increased; but it would be difficult to prove that there are not other forms of iron which act as favorably as the iodide in cases of scrofula. If that impression be correct, as much, if not more, of the benefit may be owing to the iron as to the iodine. However this may be, I have found the iodide of iron an useful tonic in such cases; and I always give it in the form of syrup, never exceeding four grains of the medicine in twenty-four hours. Whatever form of the medicine may be used,

I doubt the prudence of exhibiting it beyond a fortnight or three weeks at a time; at the end of that time, the preparation should be set aside, aperient medicines should be employed, and its use should be resumed with the same precautions. In this way any virtues which the medicine possesses are more surely brought out, and the inconveniences sometimes experienced from its administration will be, as nearly as possible, avoided.

